# Pharmacoeconomic perspectives on HIV: integrating data for long-term treatment and prevention

**DOI:** 10.3389/fmed.2025.1716965

**Published:** 2026-01-12

**Authors:** Mohd Mursal, Ghizal Fatima, Yulia Sh. Gushchina

**Affiliations:** 1 Chronobiology Unit, Department of Biotechnology, Era University, Lucknow, India; 2 Department of General and Clinical Pharmacology, Medical Institute, Peoples’ Friendship University of Russia (RUDN University), Moscow, Russia

**Keywords:** antiretroviral therapy (ART), DALY, HIV, pharmacoeconomics, PrEP, QALY, UNAIDS

## Abstract

Sustained progress against HIV has been enabled largely by antiretroviral therapy (ART), yet gaps in prevention, treatment coverage, and financing continue to threaten long-term control of the epidemic. Pharmacoeconomics provides a crucial framework for identifying interventions that deliver the greatest health gains per dollar spent, particularly in resource-limited settings where HIV programs must balance lifelong treatment needs with expanding prevention options. Economic evaluations consistently show that high-impact interventions such as dolutegravir-based ART, targeted PrEP delivery, and voluntary medical male circumcision often fall within favorable cost-effectiveness thresholds, with typical incremental cost-effectiveness ratios (ICERs) ranging from as low as US $245 per DALY averted for PrEP in serodiscordant couples to US $174–2,800 per infection averted for VMMC. This review synthesizes the pharmacoeconomic evidence across treatment and prevention, highlights the value of integrating real-world costs and equity considerations, and underscores the need for robust, context-specific economic models to support sustainable HIV responses. Achieving long-term epidemic control will depend on addressing persistent indirect costs, improving access for marginalized populations, and aligning investments with interventions that demonstrate both clinical effectiveness and compelling economic value.

## 1 Introduction

**FIGURE 1:**
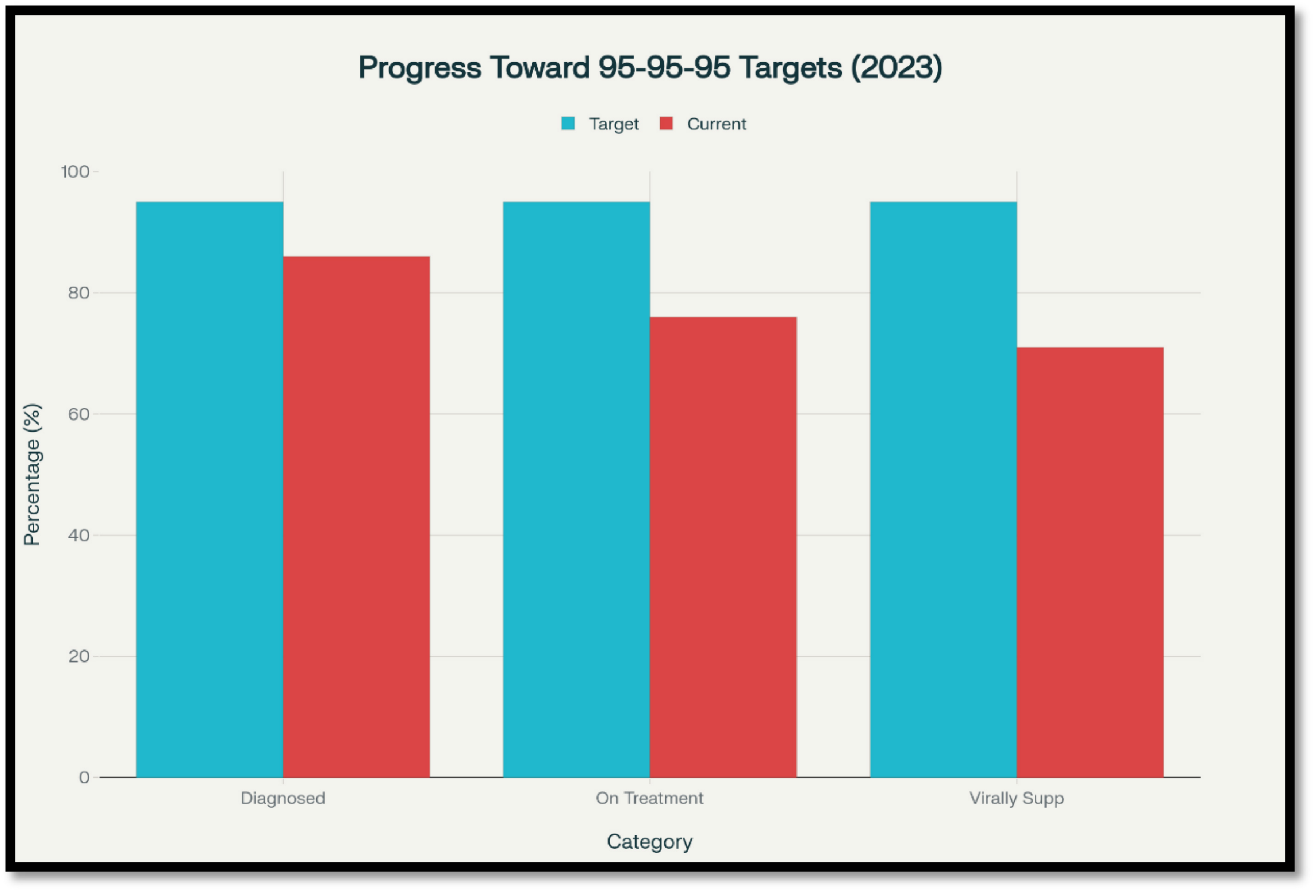
Progress toward UNAIDS 95-95-95 targets globally (2023), showing gaps in diagnosis, antiretroviral therapy (ART) coverage, and viral suppression. Source: UNAIDS Fact Sheet 2024.

Human immunodeficiency virus (HIV) remains one of the most significant health and socioeconomic challenges of the 21st century. As of the latest data available from UNAIDS, there were nearly 39.9 million individuals living with HIV at the close of 2023, with about 1.3 million new HIV infections and 630,000 AIDS-related deaths in that year alone. Pharmacoeconomics is the application of economic principles to the evaluation of pharmaceuticals and health interventions, examining both their costs and their clinical outcomes. By determining whether interventions provide sufficient value for the resources invested, it’s also helps to guide the efficient allocation of limited healthcare budgets. This framework is particularly relevant to HIV, where lifelong ART, emerging prevention technologies, and evolving service delivery strategies compete for finite financial resources. Despite significant scientific and programmatic progress that has turned HIV into a manageable chronic disease from an ultimately fatal one, the global response continues to be hampered by enormous challenges surrounding prevention, treatment coverage, and financial sustainability. Widespread roll-out of antiretroviral therapy (ART) has been a triumph of public health that has allowed millions of individuals to sustain viral suppression and significantly decrease morbidity and mortality due to HIV. As of 2023, approximately 30.7 million individuals were on ART across the world, a significant surge from 7.7 million in 2010. Yet, treatment gaps remain, especially among children only 57% of whom are on ART and among populations in resource-constrained settings. Sustained ART coverage cannot be achieved by merely having good healthcare infrastructure but also through substantial and ongoing financial commitment. At the heart of the worldwide strategy for ending the AIDS epidemic is the 95-95-95 goal, which seeks that 95% of individuals living with HIV are aware that they have HIV, 95% of those who know they have HIV are on ART, and 95% of those on treatment have viral suppression. As of 2023, global progress toward the 95-95-95 targets stood at approximately 86% of people living with HIV aware of their status, 76% of those diagnosed on ART, and 71% of those on ART achieving viral suppression, (Figure 1) underscoring persistent gaps in treatment and monitoring (1). Pharmacoeconomics, being a specialized discipline in the health economics family, is an important key to facilitating decision-making for HIV programs. Through structured assessment of the cost and outcome of drug treatment and preventive interventions, pharmacoeconomic studies inform the decision of whether new or current interventions are good value for money. This is particularly crucial in HIV, where long-term treatment regimens, novel prevention approaches [including (PrEP) pre-exposure prophylaxis and long-acting injectables], and monitoring interventions compete for finite healthcare resources (2).

Economic evaluations such as cost-effectiveness analysis (CEA), cost-utility analysis (CUA), and BIA are essential tools for policymakers. For instance, studies have shown that integrase inhibitor–based regimens, such as those containing dolutegravir, though sometimes higher in upfront cost, are often more cost-effective over the long term due to superior efficacy, better tolerability, and lower rates of resistance (3, 4). Similarly, prevention interventions like PrEP have demonstrated favorable cost-effectiveness profiles, particularly in high-incidence settings, by averting new infections and reducing future treatment costs. Despite substantial global investments, UNAIDS estimated that approximately US $20.8 billion was available worldwide in 2022, still significantly short of the US $29.3 billion annually required to meet the 2025 interim targets for epidemic control. This funding shortfall highlights the urgent need to prioritize interventions that offer the highest health gains per dollar spent, especially in settings with constrained budgets. In this economic assessment, evidence was identified through targeted searches of PubMed, Scopus, Google Scholar, UNAIDS reports, and major global HIV economic evaluations. Priority was given to studies published between 2010 and 2025, with additional inclusion of influential earlier analyses where relevant. Selection emphasized peer-reviewed cost-effectiveness, cost-utility, and budget impact studies, along with modeling analyses directly informing HIV prevention and treatment policy. we explore the core principles of pharmaco-economics as applied to HIV care and prevention, summarize key findings from global and regional economic evaluations, and discuss implications for health policy and program planning. By integrating economic evidence into HIV strategies, stakeholders can optimize the impact of available resources, support equitable access to care, and advance progress toward ending the HIV epidemic worldwide.

## 2 Pharmacoeconomic principles in HIV treatment

Pharmacoeconomics is a discipline of utmost importance that informs decision-making in healthcare through an evaluation of the value of medical interventions against their costs. In HIV, where treatment and prevention are lifelong interventions, pharmacoeconomic analysis is particularly relevant to maximize resource utilization and make care globally accessible on a sustainable basis.

Four types of pharmacoeconomic analyses are widely used: cost-minimization analysis (CMA), CEA, CUA, and cost-benefit analysis (CBA). CMA is used to compare the cost of interventions that are thought to have the same clinical outcome. For instance, comparison studies involving various generic formulations of antiretrovirals tend to utilize CMA where efficacy or safety profiles are comparable and only the least expensive option needs to be determined (5). CEA compares the cost per natural unit of health benefit obtained, like life-years gained or infections prevented. In HIV studies, CEA is often utilized to compare and contrast various ART regimens or prevention interventions, figuring out which yields the greatest health benefit for each monetary unit expended (6, 7). CUA is an extension of CEA that includes quality-of-life adjustments, frequently in the form of quality-adjusted life years (QALYs) or disability-adjusted life years (DALYs). CUA is especially important in HIV due to its consideration of the long-term nature of the disease and the long-term effects of ART side effects. Dolutegravir-containing regimens, for occurrence, have shown cost-utility benefits relative to other regimens in a variety of settings (8, 9). CBA converts health effects into dollars and allows a direct economic comparison of costs and benefits. Although less frequently employed concerning HIV, CBA is useful for assessing large-scale public health interventions, for example, national prevention programs or testing programs (10). Of these strategies, CUA has been regarded as the most informative and is commonly used in HIV pharmacoeconomic research as it captures survival and improvements in quality of life (Table 1). Furthermore, analysis of incremental cost-effectiveness ratios (ICERs) or the incremental cost for the additional QALY gained is a conventional measure used to ascertain whether an intervention is cost-effective relative to willingness-to-pay thresholds (11). The World Health Organization recommends that interventions with an ICER less than the per capita GDP per QALY are very cost-effective. Pharmacoeconomic modeling in HIV also relies on certain modeling methodologies, such as Markov models and dynamic transmission models, which enable the simulation of long-term results and indirect effects like decreased transmission (12). Leveraging these analyses in policy assists in prioritizing regimens and interventions that offer the greatest value for money, improving, in the end, patient and public health outcomes internationally.

**TABLE 1:** Types of pharmacoeconomic analyses commonly used in HIV-related evaluations, with selected applications and examples from recent literature.

Analysis type	Definition	Outcome measure	Use in HIV	Example	Advantages	Limitations
Cost-minimization (CMA)	Compares cost of equally effective interventions	$ only	Comparing bioequivalent generics	TDF vs. tenofovir alafenamide (when efficacy same)	Simple	Only when outcomes equal
Cost-effectiveness (CEA)	Cost per clinical benefit	$/life-year saved	ART regimens, VMMC	DTG-based ART vs. EFV in South Africa	Easy to apply	Ignores QoL
Cost-utility (CUA)	Cost per QALY/DALY	$/QALY or DALY	ART and PrEP	PrEP in Thailand	Captures QoL and survival	Data intensive
Cost-benefit (CBA)	Compares benefits and costs in $	Net monetary value	Testing campaigns	National HIV awareness program in USA	Converts to policy-friendly terms	Subjective valuation of health
Budget impact analysis (BIA)	Evaluates total cost of implementation	$/program or $/population	PrEP, DTG switch	VMMC scale-up in South Africa	Real-world planning tool	Requires national data
Distributional CEA	Cost-effectiveness + equity	ICER + equity weight	Targeting FSW, MSM, adolescents	PrEP for AGYW vs. SDC	Considers equity	Not yet standardized
Cost-consequence	Lists all outcomes separately	Mixed units	New technologies	Long-acting injectable ART	Flexible	Hard to summarize

### 2.1 Critical evaluation of pharmacoeconomic modeling approaches in HIV

Economic evaluations in HIV care heavily rely on mathematical modeling, and while these approaches provide valuable projections, they also introduce methodological constraints that affect the interpretation of cost-effectiveness metrics. Most studies employ Markov state-transition models, which simulate disease progression through predefined health states but cannot capture feedback loops, behavior change, or reductions in transmission resulting from successful treatment or prevention coverage. As a result, Markov models may underestimate the broader population-level benefits of interventions such as PrEP, TasP, or VMMC (voluntary medical male circumcision). In contrast, dynamic transmission models incorporate secondary infections averted and account for shifts in incidence attributable to growing coverage, but they require stronger assumptions about sexual networks, mixing patterns, and contact rates. These assumptions vary widely across studies, contributing to heterogeneity in ICERs and limiting comparability across settings.

A key limitation in the current literature is the heavy reliance on ICERs without adequately interrogating the assumptions underlying them, such as baseline incidence, adherence levels, drug pricing, and discount rates. Because ICERs are highly sensitive to epidemiological context, interventions considered cost-effective in high-incidence settings may not meet willingness-to-pay thresholds in regions with lower transmission rates. Similarly, time horizon selection substantially affects results: short time horizons tend to undervalue prevention interventions whose benefits accumulate over decades, while lifetime horizons may overestimate real-world feasibility in settings with unstable financing or limited implementation capacity. Sensitivity analyses are often performed, yet many evaluations focus on univariate rather than probabilistic sensitivity analyses, limiting insight into overall uncertainty. Parameters such as adherence, PrEP persistence, treatment failure rates, drug resistance emergence, and unit costs exert outsized influence on ICER outcomes but are inconsistently reported. As a result, policymakers may overinterpret point estimates without fully appreciating the range of plausible economic outcomes. More transparent reporting of assumptions, clearer justification for modeling choices, and standardized sensitivity analysis frameworks would enhance methodological rigor and facilitate cross-study comparison.

## 3 Cost drivers in HIV management

The management of HIV infection involves complex, lifelong interventions that require substantial financial commitment. While advances in treatment have improved clinical outcomes and transformed HIV into a manageable chronic disease, the economic burden remains significant and multifaceted. Understanding the primary cost drivers is essential to inform policies aimed at optimizing resource utilization and achieving sustainable care delivery, particularly in low- and middle-income countries (LMICs) (13).

**FIGURE 2:**
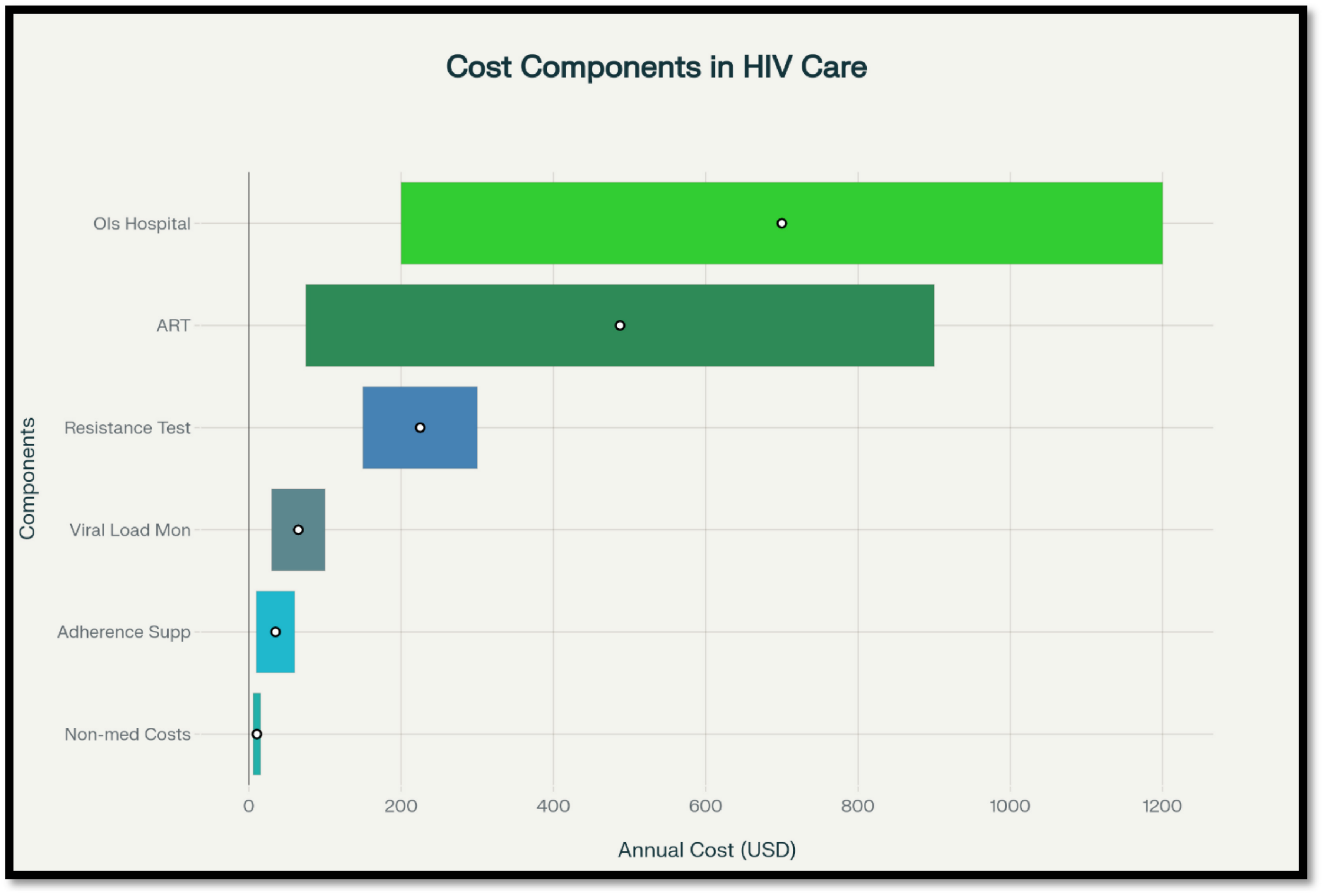
Annual cost ranges for major components of human immunodeficiency virus (HIV) care across different healthcare settings.

Antiretroviral therapy remains the dominant cost component, accounting for 60%–70% of lifetime HIV care costs in many settings. Even though generics have reduced prices in LMICs, annual ART expenditures still represent the largest share of national HIV budgets. Integrase inhibitor–based first-line regimens, especially dolutegravir (DTG), have higher upfront costs but are frequently Cost effective because of superior viral suppression, lower resistance, and fewer switches to expensive second-line therapies (Figure 2).

In LMICs, generic ART expansion has lowered overall costs, yet recent facility-level data from African settings still report annual treatment expenses of US $136–278 per patient, reflecting both clinical care and support services. Despite these reductions, ART remains a major budget burden due to the rising number of people requiring lifelong therapy. Newer INSTI-based regimens, particularly dolutegravir, offer superior virological efficacy, improved resistance profiles, and better tolerability compared to older NNRTI-based options (14–16). Although initially more expensive, modeling studies consistently show long-term cost savings by reducing treatment failure and minimizing progression to second-line and salvage therapies. A large South African economic model projected cumulative savings of about US $300 million over ten years following widespread adoption of dolutegravir-based first-line ART, driven by improved adherence and fewer resistance-related switches. Additionally, regimen simplification through single-tablet combinations enhances adherence and patient satisfaction, indirectly lowering long-term costs by reducing virologic failure and hospitalization (17–19).

Adherence and retention services, including community-based differentiated service delivery, digital adherence tools, and pharmacist-led counseling, introduce additional program costs but are consistently cost-effective. These interventions reduce virologic failure, hospitalizations, and the need for second-line regimens, yielding downstream savings (20, 21).

Laboratory monitoring, especially viral load testing, is another significant cost contributor. Central laboratory testing remains expensive in many LMICs, whereas point-of-care viral load systems have shown favorable cost-effectiveness by improving treatment monitoring and preventing prolonged failure. Routine resistance testing remains limited in resource-constrained settings due to cost, though its absence can increase long-term expenditures through avoidable regimen switches (22, 23). Opportunistic infection–related hospitalizations impose substantial financial burden, often exceeding annual outpatient ART costs. Early ART initiation, cotrimoxazole prophylaxis, and targeted screening (e.g., for cryptococcal antigenemia) demonstrate strong economic value by preventing high-cost inpatient care (24–26).

### 3.1 Indirect and societal costs

Beyond medical expenditures, the economic burden of HIV encompasses productivity losses, out-of-pocket spending, transportation costs, and caregiver responsibilities. These indirect costs are often overlooked in policy debates yet have substantial consequences, particularly in LMICs where many affected individuals are in their most economically productive years (27). In Tanzania and Uganda, non-medical costs averaged US $7.33 per clinic visit, representing a considerable share of household income. Similarly, a modeling study from Mexico estimated a 5-year societal cost of US $8,432 per patient, with an ICER of US $2,218 per QALY gained—well within national willingness-to-pay thresholds. Interventions such as multi-month dispensing, decentralized drug distribution, and differentiated service delivery can significantly reduce these indirect costs while improving adherence and patient satisfaction (27–29) (Table 2). Global variability in cost drivers reflects differences in drug pricing, health-system capacity, financing mechanisms, and local epidemiology. In high-income countries, ART drug expenditures and laboratory monitoring dominate overall costs, whereas in many LMICs, non-medical expenses and hospitalization contribute a larger proportion of total burden. Consequently, resource-allocation strategies must be tailored to national contexts, ensuring that investments address both immediate clinical needs and longer-term societal impacts. Integrating direct and indirect cost components into pharmacoeconomic evaluations is essential for guiding sustainable program design and ensuring equitable access to effective HIV care worldwide (30, 31). These findings can be summarized through the following key points:

• ART represents the largest share of HIV care costs globally.
• DTG-based first-line regimens are often Cost effective due to reduced treatment failures.
• Adherence interventions add small upfront costs but prevent expensive downstream failures.
• Point-of-care viral load testing improves outcomes and is highly cost-effective.
• Preventing opportunistic infections significantly reduces hospitalization costs.
• Non-medical and societal costs are substantial and should be included in economic planning.

**TABLE 2:** Major cost components in human immunodeficiency virus (HIV) care and treatment across various program settings, including antiretroviral therapy (ART), monitoring, and adherence-related expenditures.

Cost driver	Components	Regional settings	Annual cost range	Notes
ART	Drug costs, stock management, procurement	South Africa, Malawi, USA	$75–$900	Cost drops with generics
Adherence	CHW, DSD, SMS reminders	Pakistan, Kenya, Uganda	$10–$60/patient/year	Linked to better outcomes
Viral load monitoring	Central lab, point-of-care	India, Mozambique	$30–$100/test	POC more cost-effective long-term
Resistance testing	Genotyping, sequencing	Nigeria, Vietnam	$150$300/test	Often skipped in LMICs
Ois and hospitalization	TB, CMV, Cryptococcus	SSA hospitals	$200–$1,200/episode	Often preventable
Clinic visits	Consultation, counseling	Tanzania	∼$7/visit	Frequent visits add burden
Non-medical Costs	Transport, lost income	Uganda, Lesotho	$6–$15/visit	Often > 20% of household income
Human resources	Salaries, training	Global	20%–30% of budget	Shortages affect efficiency
Pediatric ART	Child-specific drugs, weights	Sub-Saharan Africa	$120–$500/year	Lower access than adults
Supply chain	Logistics, cold storage	LMICs	∼5%–10% of total ART budget	Often underfunded
Long-acting ART	Injection cost, clinic time	Pilot programs	TBD	Promising but costly

## 4 Cost-effectiveness of HIV prevention strategies

**FIGURE 3:**
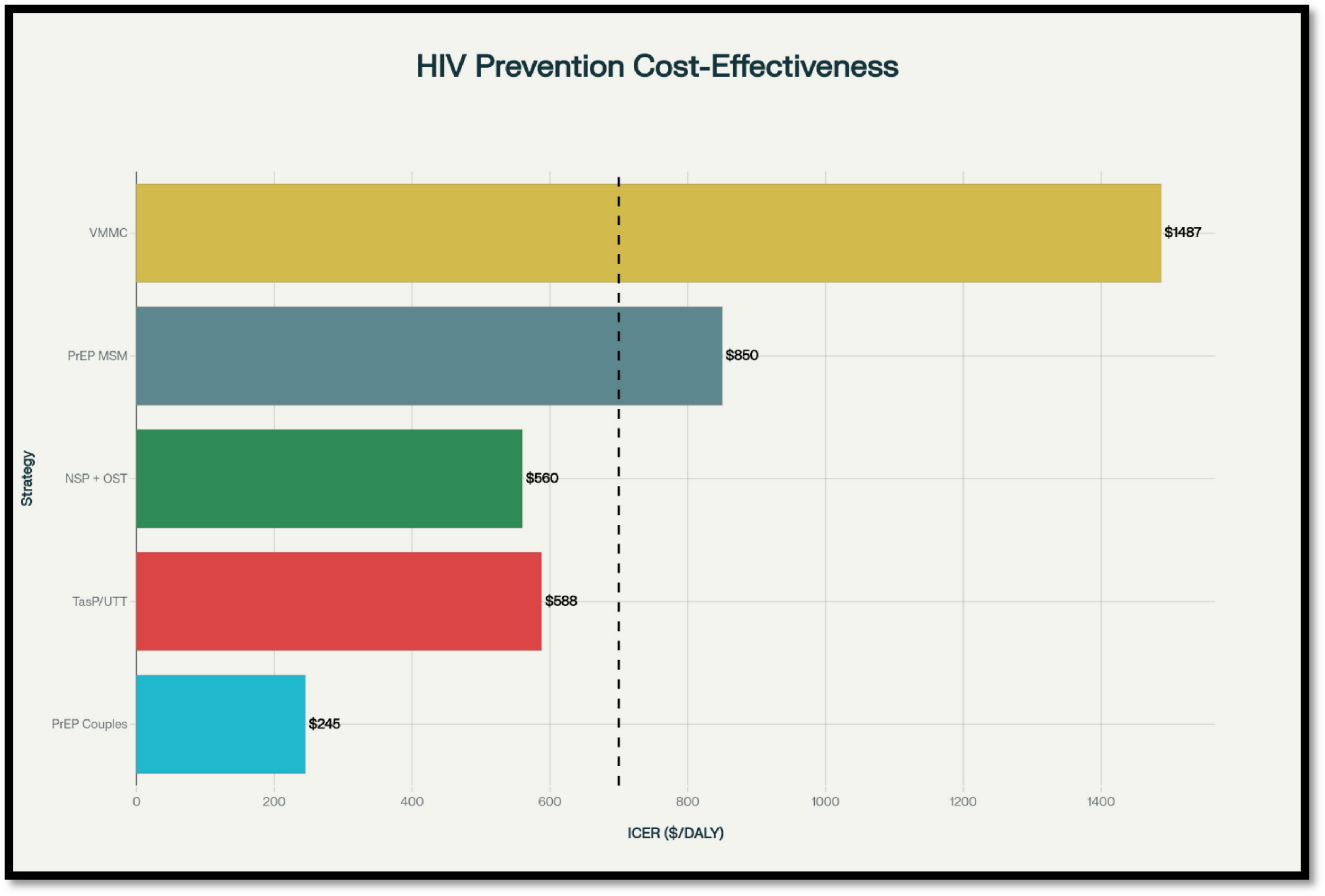
Incremental cost-effectiveness ratios (ICERs) of major human immunodeficiency virus (HIV) prevention interventions, showing disability-adjusted life years (DALYs) averted across diverse target populations.

Human immunodeficiency virus prevention is the most powerful tool to reduce long-term health and economic burdens. Evaluating cost-effectiveness helps guide policymakers on which interventions provide the most value for money. Below, we examine the evidence for major prevention strategies in detail (Figure 3). To support comparability across interventions, a quantitative synthesis table summarizing typical ICER ranges, time horizons, and main cost drivers is provided below in Table 3.

**TABLE 3:** Incremental cost-effectiveness ratio (ICER) values represent typical ranges reported across multiple economic evaluations in low-, middle-, and high-income settings.

Intervention	Target population	Setting	Typical ICER (QALY/DALY)	Time horizon	Main cost drivers
PrEP (Serodiscordant couples)	SDC	Kenya/SSA	∼US$245 per DALY	Lifetime	Drug cost, adherence, targeting efficiency
PrEP (MSM)	MSM	Thailand/Kenya/HIC	∼US$850 per DALY/QALY	Multi-decade	Incidence, drug price, delivery model
PrEP (AGYW)	AGYW	East/Southern Africa	∼US$12,351 per DALY	10–30 years	Coverage scale, program cost, retention
VMMC	Males 15–49	Eastern/Southern Africa	US$174–2,808 per infection averted	Lifetime	Unit cost, prevalence, age targeted
Condom distribution	General/high-risk groups	Global	Very low; consistently cost-effective	Short–medium	Procurement, distribution, adherence
TasP/UTT	All PLHIV	SSA	∼US$530–645 per DALY	Long-term	ART cost, testing/linkage, suppression rates
NSP + OST	PWID	Eastern Europe/Asia	US$210–910 per life-year	5–15 years	Service integration, coverage, OST access
First-line: DTG vs. EFV	ART-naïve adults	LMICs	Cost effective or dominant	10–20 years	Drug cost, failure rate, switch prevention
Second/third-line regimens	ART-experienced/resistant	Global	High; often > US$1,500–2,000 per patient-year	Medium–long	Drug procurement, monitoring, hospitalization

Estimates vary based on incidence levels, drug pricing, adherence, delivery models, and modeling assumptions. DALY, disability-adjusted life year; QALY, quality-adjusted life year; PWID, people who inject drugs; UTT, universal test-and-treat; TasP, treatment as prevention; VMMC, voluntary medical male circumcision; AGYW, adolescent girls and young women; SDC, serodiscordant couples.

### 4.1 Pre-exposure prophylaxis

High-quality models show PrEP can be cost-effective when targeted to high-risk groups, but results vary by setting and strategy. For example, in western Kenya a modeling study found that PrEP for women with multiple partners cost ∼$1,898–2,351/DALY, while broad rollout to all adolescent girls/young women (AGYW) was much higher (∼$12,351/DALY). These results highlight that targeted PrEP (e.g., to SDCs, FSWs, high-risk MSM) is far more efficient than untargeted campaigns (32–34).

In Sub-Saharan Africa, a Kenyan modeling study in six counties estimated that PrEP would prevent 4.5%–21.3% of new HIV infections. The incremental cost-effectiveness ratios (ICERs) ranged from as low as $245 per disability-adjusted life year (DALY) prevented for serodiscordant couples to as much as around $1,900–$2,400 per DALY for other high-risk groups. These results strongly indicate that targeted PrEP interventions, specifically among serodiscordant couples, are much more cost-effective than larger programs targeting adolescent girls and young women (AGYW) (32, 35–37).

In Southeast Asia, Thailand evidence reported in The Lancet assessed various PrEP delivery models among men who have sex with men (MSM). It identified that a key-population led delivery model of PrEP was most cost-effective, with ICERs between 28,000 and 37,300 Thai Baht per quality-adjusted life year (QALY) gained (or about USD $850–1,200 per QALY) (38). Additionally, this strategy had the greatest epidemiological impact, averting approximately 58% of new HIV infections among MSM. In the high-income setting, a prominent United Kingdom analysis [Phillips et al. (39)] modeled an event-based PrEP intervention among MSM. Scaling from 4,000 first-year initiations to 40,000 in year 15, the model estimated substantial outcomes: around €1.0 billion in net cost savings, a 25% decrease in new infections, and around 40,000 QALYs gained over the 80-year horizon. More generally, systematic reviews report wide variability in PrEP cost-effectiveness, from Cost effective to about $39,900 per infection averted. PrEP is most beneficial in high-incidence environments or where drug prices are lower, but it’s worth decreases in lower-incidence environments or with high drug costs (40–42).

Overall, recent studies (post-2018) consistently show PrEP can be cost-effective or even Cost effective for key populations in Africa and Asia. In high-income countries, PrEP is also economically attractive among MSM with ongoing transmission. Cost-effectiveness often hinges on incidence, targeting efficiency, and drug prices (Table 4) (43).

**TABLE 4:** Comparative cost-effectiveness of key human immunodeficiency virus (HIV) prevention strategies across different target populations and regions.

Strategy	Target group	Country	ICER
Oral PrEP - MSM	MSM	Kenya	$850 per DALY
PrEP - Serodiscordant Couples	SDC	Kenya	$245 per DALY
PrEP - AGYW	Young women	Kenya	$12,351 per DALY
VMMC	Males 15–49	SSA	$174–$2,800/infection averted
TasP (UTT)	All PLHIV	Zambia, SA	$530–$645 per DALY
NSP + OST	PWID	Belarus, Georgia	$210–$910/life-year

### 4.2 Voluntary medical male circumcision (VMMC)

Multiple economic analyses demonstrate that VMMC is a highly cost-effective HIV prevention intervention in high-prevalence settings and can generate substantial long-term savings for health systems. One systematic analysis estimated that VMMC costs between US$174 and US$2,808 per HIV infection averted in sub-Saharan Africa, highlighting its strong economic value in regions with generalized epidemics (43, 44). Country-specific model-based studies have also indicated robust economic value, often indicating net Cost effective negative incremental cost-effectiveness ratios (ICERs). In South Africa, a 2016 PLOS One modeling study projected VMMC as Cost effective in all provinces except Gauteng, even at a unit cost of up to $225 (45). The study concluded that VMMC would decrease total healthcare costs by avoiding new infections and future ART costs in eight out of nine provinces, with considerable benefits at the national level in the long term. Outside of Africa, data from the Avahan HIV prevention program in India targeting female sex workers (FSWs) showed that male circumcision was one of the most cost-effective interventions at a cost of less than $400 per infection averted [Nachega et al. (46)]. A wider Lancet review across sub-Saharan Africa reported VMMC ICERs of around $442–$4,096 per infection averted, varying by country setting. In contrast, (47) Njeuhmeli et al. highlighted net savings overall from circumcision in high-prevalence countries.

More recent global analyses support these results. A 2023 UNAIDS press release presenting Lancet Global Health modeling reported that sustaining VMMC programs to 2028 would be Cost effective or cost-effective in almost all sub-Saharan Africa scenarios (48). Even at low-incidence levels (<1% incidence), VMMC was cost-effective in 62% of the modeled scenarios, and at high-incidence levels (>10%), it was cost-effective in 95% of scenarios. One South African study also showed that circumcising 20-year-old men would be a 14.5% lifetime return on investment, or about $617 in discounted healthcare costs per circumcision. Overall, VMMC is a high-impact, cost-effective intervention in various African contexts. Modeling repeatedly affirms that it can prevent infections at a low cost with net savings by lowering future ART costs. Strong evidence heavily reinforces continued and intensified scale-up of VMMC, especially in high-prevalence areas (49–51).

### 4.3 Condom promotion and distribution

Condom distribution and promotion programs carefully integrating awareness campaigns with free or subsidized condom availability are generally understood to be cost-effective prevention interventions for HIV, although evidence from settings is somewhat mixed. Modeling analyses of sub-Saharan African female condom distribution have had notably positive cost-effectiveness profiles. For instance, one analysis of 13 high-prevalence countries estimated that distributing 100,000 “Woman’s Condoms” each country per year would prevent an estimated 21 HIV infections yearly, being cost-effective according to WHO-CHOICE criteria. Another review noted that female condom interventions cost as low as $107–$303 per disability-adjusted life year (DALY) prevented, with examples stretching from Zimbabwe to Mozambique far below traditional cost-effectiveness thresholds (52). Focused condom promotion among core populations has also demonstrated robust economic worth. In Peru, a modeling analysis of transgender female sex workers estimated that a full prevention package involving promotion of condoms with clients and partners, increased HIV testing, and other services would prevent almost 50% of new infections over 10 years. At a cost of approximately $816,000 per year (about 2% of the Peruvian national HIV budget), this intervention had a cost per DALY averted of less than $1,300, indicating cost-effectiveness in this setting (53–55).

In a similar way, a programmatic data analysis from Nigeria (within Avahan evaluation) revealed that condom provision to female sex workers (FSWs) and their clients was one of the most cost-effective approaches, although the exact ICER values differed by district. On balance, modeling and evidence indicate that condom promotion and availability are “high-value” interventions, especially if targeted at high-risk groups (56, 57). Overall estimates for the cost of preventing a single HIV infection through condom programs range from around $550 to $2,240, varying with the setting and compliance levels. Although regular use is still a key challenge, condoms are still cheap, readily available, and very effective when used correctly. WHO and UNAIDS still highly recommend mass distribution of condoms as a key part of overall HIV prevention, particularly for key populations. Serious monitoring and evaluation are still necessary to maximize program efficiency and maintain impact (58).

### 4.4 Treatment as prevention (TasP)

Scaling up antiretroviral therapy (ART) to all HIV-positive patients indiscriminately, known as “universal test-and-treat” (UTT), has been proven to be extremely cost-effective in various settings. Significantly, simulation using the HPTN 052 trial in serodiscordant couples illustrated that initiating ART early (rather than delayed) was Cost effective in South Africa with a 5-year time horizon based on the prevention of opportunistic infections. Per lifetime, early ART provided an extremely low cost of about $590 per life-year gained. In India, comparable analyses determined early ART to be cost-effective, at about $1,800 per life-year gained at 5 years, falling to about $530 per lifetime. These results highlighted individual health gains and notable public health benefits, creating strong grounds for immediate treatment of HIV-positive partners (59, 60).

Evidence from community-level UTT trials further supports these conclusions. A 2021 modeling analysis based on data from the PopART (HPTN 071) trial communities in Zambia and South Africa projected incremental cost-effectiveness ratios (ICERs) of approximately $593 per DALY averted in Zambia and $645 in South Africa (61, 62). On a per-infection basis for the 2014–2017 period, costs were estimated at $1,318 and $2,236, respectively. At standard cost-effectiveness thresholds (∼$700–$800 per DALY), UTT had over a 95% probability of being considered cost-effective. These data align with WHO’s 2015 “Treat All” guidelines and the Disease Control Priorities (DCP3) framework, which consistently rank treatment as prevention (TasP) among the most cost-effective strategies in high-burden settings, with cost per DALY often below $100 when accounting for averted morbidity and mortality (61, 63, 64).

While UTT entails substantial upfront costs, long-term savings from prevented HIV cases and reduced morbidity typically offset these expenditures. For example, the PopART program projections estimated annual implementation costs of approximately $6–$8 per person (2014–2017), a modest figure relative to the lifetime costs of ART. Nonetheless, careful financial planning is required to ensure sustained scale-up, as emphasized by UNAIDS and WHO. Overall, UTT emerges as a highly impactful and economically justified intervention for controlling HIV epidemics in high-prevalence regions.

### 4.5 Needle and syringe programs (NSPs)

Needle and syringe programs, along with other harm reduction services for injecting drug users (IDUs), are consistently found to be highly cost-effective and often even Cost effective. In the US, an early economic estimate indicated that providing sterile syringes for all injections costs about $34,300 per HIV infection prevented. Since the lifetime medical expense of one case of HIV far surpasses this figure, bulk syringe access programs were estimated to leave the healthcare system with net savings. While ICERs might rise at high levels of coverage, overall societal savings from widespread syringe access are large. In Central Asia and Europe, modeling analyses also buttress these findings. A review of Belarus, Georgia, Kazakhstan, Moldova, and Tajikistan estimated that scaling up NSPs, particularly in the presence of OST and ART, generated very favorable ICERs. For example, integrating NSP and OST (Strategy 8) was classified as “very cost-effective” in Belarus and Georgia and had an estimated ICER ranging from $210 to $910 per life-year gained. More intensive intervention packages, which included NSP, OST, ART, and other services, were always Cost effective (frequently under $1,000–$2,000 per life-year saved) and even cost-saving in Kazakhstan. From a policy viewpoint, these results highlight the economic value of NSPs, which generally attain costs ranging from $100 to $1,000 per HIV infection prevented, far below globally accepted thresholds of cost-effectiveness. Evidence invariably shows that even small coverage increases in NSP resulted in dramatic declines in HIV and HCV transmission among PWID. In reality, NSPs are increasingly accepted as “best buys” for PWID populations in HIV prevention. They are not that costly, offer several benefits to health beyond HIV (also including HCV prevention), and can be delivered through a variety of delivery models like fixed locations, pharmacies, and mobile outreach. Evidence from modeling reinforces widespread coverage of NSPs especially combined with OST as having high impact and economic value public health intervention in various regions (65).

## 5 Cost-effectiveness of different ART regimens

Economic analyses of antiretroviral therapy (ART) regimens are important for policy and resource planning in HIV programs worldwide. First-line ART regimens, comprised of two nucleoside reverse transcriptase inhibitors (NRTIs) and a third drug, have been generally accepted as the most Cost effective choice because they are less expensive, easier to administer, and have greater initial efficacy. In the sub-Saharan region of Africa, first-line regimens have been approximated at $137–$320 per patient annually, whereas second-line regimens, commonly protease inhibitor (PI)-based, cost twice or three times as much due to the necessity for more costly boosted drugs and more intense monitoring (66). Third-line or salvage therapy is reserved for drug-resistant patients and may cost as much as $1,000–$2,000 annually (67).

**FIGURE 4:**
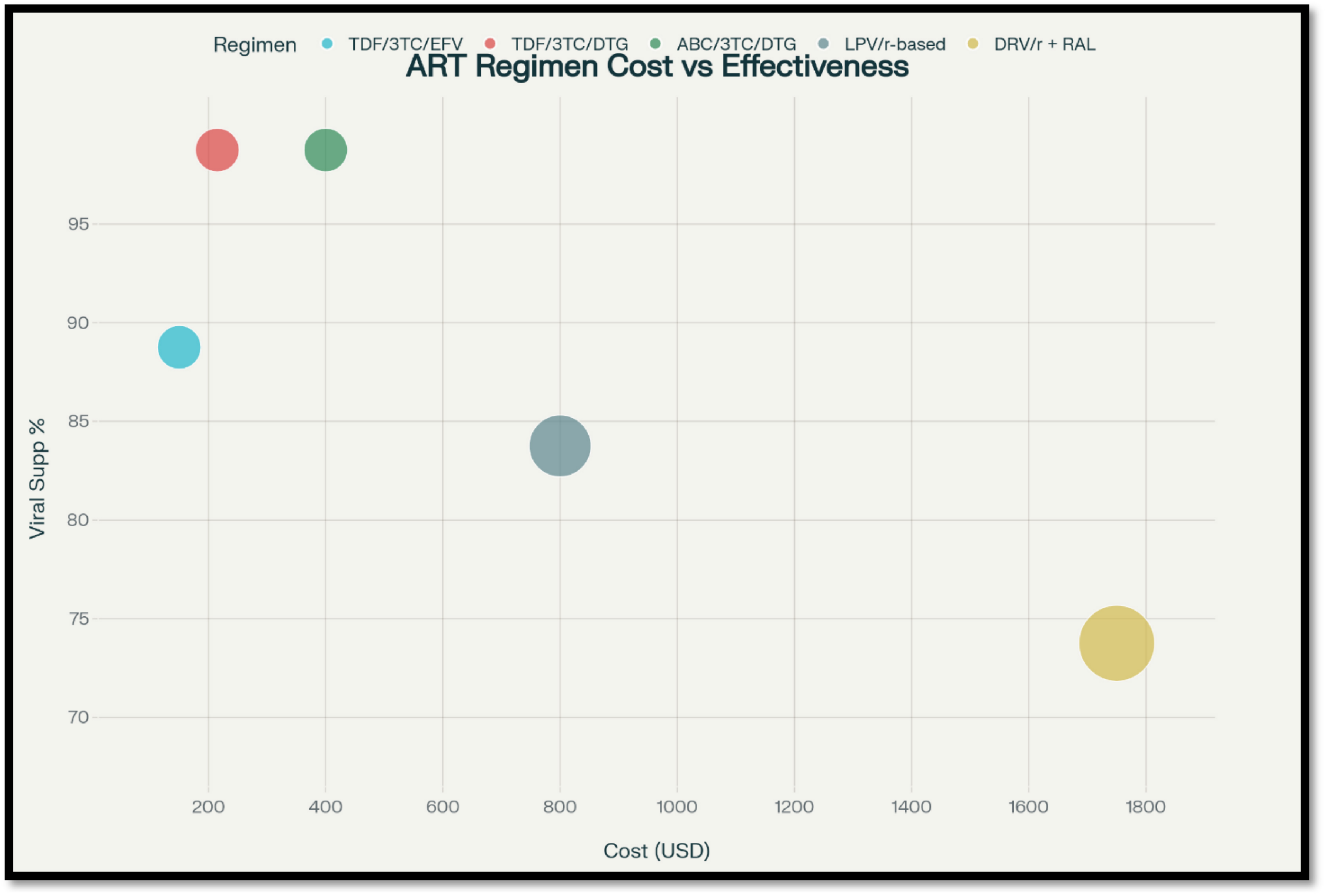
Comparison of antiretroviral therapy (ART) regimens showing relationship between cost, effectiveness, and treatment line.

The use of Integrase strand transfer Inhibitors (INSTIs), more so dolutegravir (DTG), has transformed first-line treatment practices across the globe. Dolutegravir provides greater potency, fast viral suppression, and a more prominent barrier to resistance in comparison to NNRTIs and Pis. South African modeling studies presented evidence that DTG-based regimens not only enhanced clinical response but also led to long-term cost savings from reduced treatment failures and lowered switching rates to costly second-line regimens. The South African ADVANCE trial further corroborated the findings, presenting superior efficacy and non-inferior safety profiles for DTG-based regimens versus EFV-based therapies (Figure 4) (68–70).

Analyses in Uganda and Malawi concluded that adding DTG to first-line ART was very cost-effective, with incremental cost-effectiveness ratios (ICERs) well below $500 per life-year gained, well below WHO levels for cost-effectiveness (39, 71). In a major regional modeling study involving 22 low- and middle-income countries, dolutegravir-based regimens outranked NNRTI-based regimens both for health outcomes and for value for money in all-compassing analyses, facilitating its widespread use (72). Though their clinical advantages, PI-based regimens continue to be costlier and are largely reserved for second-line use after failure of first-line regimens (Table 5). A cost-effectiveness comparison showed that changing over to boosted PI regimens after NNRTI failure cost around $739–$1,399 per QALY gained, which varied according to local pricing and resistance (73). While necessary, the increased expense of these regimens highlights the need to avoid first-line failures by using strong adherence support and the choice of high-barrier agents such as dolutegravir.

**TABLE 5:** Annual cost estimates and clinical performance (viral suppression rates) of commonly used antiretroviral therapy (ART) regimens across different lines of treatment, based on recent multicenter and modeling studies.

Regimen	Line	Components	Annual cost	Viral suppression rate	Side effects	Notes
TDF/3TC/EFV	First	NNRTI-based	$100–$200	∼80%–90%	CNS effects, resistance	Older first-line
TDF/3TC/DTG	First	INSTI-based	$130–$300	>95%	Weight gain	WHO recommended
ABC/3TC/DTG	First (alternate)	INSTI-based	∼$400	>95%	Hypersensitivity	Used in children
AZT/3TC/NVP	Legacy	NNRTI-based	$75–$150	<80%	Anemia	Phased out in many LMICs
LPV/r-based	Second	PI-based	∼$800	75%–85%	GI, lipid changes	Requires cold storage
DRV/r + RAL	Third	Salvage	$1,500–$2,000	∼70%	Complex dosing	For resistance cases
CAB + RPV (injectable)	First (pilot)	Long-acting	TBD (est. ∼$1,500)	∼90%	Injection site reactions	Under study
Pediatric DTG	First	DTG dispersible	∼$150-$300	>85%	Well-tolerated	Scaling up

Includes first-line INSTI and NNRTI regimens, as well as second- and third-line salvage therapies.

Safety concerns for dolutegravir’s profile, mainly for neural tube defects in women of childbearing age, were relieved in subsequent data from the Tsepamo study in Botswana, showing a low absolute risk, prompting WHO to recommend DTG as the first-line drug of choice worldwide, including in women with adequate counseling [Fatima et al. (74), Zash et al. (75)]. The Phillips et al. modeling study projected that universal adoption of dolutegravir-based regimens across sub-Saharan Africa could prevent up to 131,000 deaths and save approximately US $860 million in treatment costs over a 20-year period, due to increased virological suppression and fewer expensive treatment switches (76). Assessments of third-line or salvage regimens are still limited because they have relatively low utilization and high expenses. These regimens are, however, important in the case of individuals with multi-drug resistance. Economic assessments note that the cost-effectiveness of these treatments significantly relies on judicious patient selection and robust support to ensure viral suppression. In general, the comparative cost-effectiveness information that highlights the importance of maximizing clinical benefits and economic value, by optimizing starting ART regimens, is reinforced by dolutegravir-based regimens, which offer better outcomes at lower long-term cost, and are reaffirming their role in attaining global targets for treatment (77, 78).

### 5.1 Modeling assumptions and uncertainty

Economic evaluations of HIV interventions rely on assumptions that introduce uncertainty into cost-effectiveness estimates. Most analyses use Markov models, which simulate transitions between clinical states but do not capture reductions in onward transmission. Dynamic transmission models incorporate population-level effects and are therefore preferred for evaluating PrEP, TasP/UTT, NSP/OST, and VMMC, though they require stronger assumptions about behavior, transmission probabilities, and intervention coverage.

Results also depend on the time horizon and discounting: longer horizons are needed to reflect lifetime benefits of prevention, while standard discounting (3%–5% annually) reduces the apparent value of interventions whose benefits accrue far into the future. Parameter uncertainty including incidence, drug prices, adherence, resistance patterns, and monitoring costs further affects ICERs, making sensitivity analyses essential for identifying influential inputs. Threshold analyses help define incidence or price levels at which interventions remain cost-effective (79).

Finally, transferability across settings is limited. Differences in drug procurement costs, delivery models, health-system capacity, and population risk profiles mean that ICERs derived in one context cannot be directly applied to another without adjustment. Recognizing these uncertainties is critical for interpreting pharmacoeconomic findings and guiding policy decisions.

## 6 BIA and long-term economic implications

**FIGURE 5:**
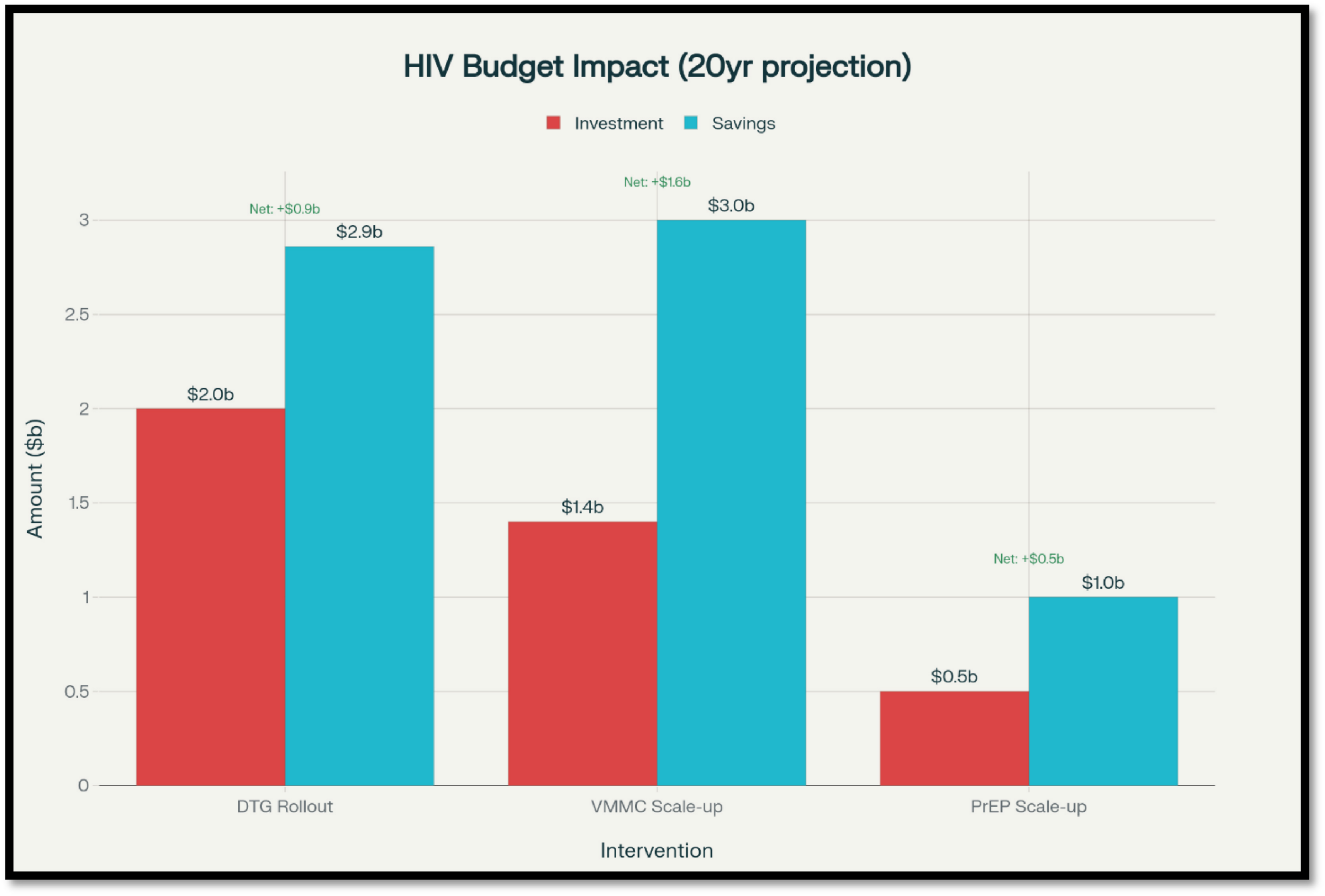
Budget impact analysis (BIA) showing long-term return on investment for major human immunodeficiency virus (HIV) interventions.

While CEA helps identify interventions that provide good value for money, budget impact analysis (BIA) assesses the financial feasibility of implementing these interventions on a large scale. BIA provides practical information to policymakers and program managers, helping them understand how scaling up new prevention and treatment strategies will affect national and donor budgets over time (Figure 5) (80).

Human immunodeficiency virus remains a major drain on health budgets worldwide, particularly in low- and middle-income countries, where donor contributions are crucial for sustaining programs. UNAIDS estimated that global resources available for HIV in 2022 totaled approximately $20.8 billion, still falling short of the $29 billion target needed to meet the 2025 interim milestones toward ending AIDS as a public health threat (81). Scaling up of such prevention interventions as PrEP, VMMC, and NSPs involves a high initial investment but can result in huge long-term cost savings through reduction in new infections and thereby future treatment expenses. For instance, in South Africa, scaling up VMMC to 80% coverage among men aged 15–49 years was estimated at an additional $1.4 billion in 15 years but would prevent over 1 million new infections, which translates into significant ART savings (45, 82). In the same manner, expanding PrEP to high-risk groups, including sex workers and MSM, entails upfront costs for drugs, laboratory testing, and adherence assistance. But studies employing models indicate that focusing PrEP on people at very high risk (e.g., HIV incidence greater than 3% per year) can generate cost savings through avoided costly lifelong ART (83). In Thailand, a 5-year budget analysis proved that increasing access to PrEP among MSM via community-based programs would cost an estimated $22 million but could prevent thousands of new infections, saving on future treatment costs (84–86). On the treatment front, the shift to dolutegravir (DTG)-based first-line regimens, while initially connoting increased drug procurement expenses in certain settings, has been estimated to save money by lowering the rate of treatment failure and the requirement for expensive second-line regimens. In sub-Saharan Africa, broad use of DTG was estimated to save up to $860 million over 20 years, avoiding more than 130,000 deaths (87). Yet budget impact also is shaped by programmatic realities. Cost of implementation, supply chain problems, and other requirements for training and community mobilization can all add to real-world expenses over and above strictly clinical cost. For instance, home-based HIV testing and universal test-and-treat (UTT) strategies, as implemented in the PopART trial conducted in Zambia and South Africa, entailed heavy up-front investments in outreach teams, logistics, and laboratory facilities. In spite of such expenses, the interventions were economically justified considering their capacity to lower HIV incidence significantly in the long term (88). In addition, long-term sustainability demands ongoing investment in health system strengthening, such as laboratory capacity for viral load measurement, strong data systems, and support services for adherence. Without ongoing investment, the full economic value of interventions cannot be expected, and resistance rates would rise, with resulting increased future costs (89).

The dynamic interplay among donor funding (e.g., Global Fund, PEPFAR), national government, and out-of-pocket spending also influences budget dynamics. In most low-income countries, dependence on outside funding ensures that changes in donor priorities can directly imperil the sustainability of prevention and treatment services. National planning, as such, needs to pay close attention to how to stabilize and diversify sources of financing in order to build a sustainable long-term program. Together, budget impact analyses emphasize that new HIV prevention and treatment approaches can be extremely cost-effective, but their scale-up depends on meticulous financial planning, phased implementation, and sustained political commitment (90, 91). By investing in strategic high-impact interventions immediately, countries can minimize future treatment burdens and thereby develop more sustainable HIV responses (Table 6) (92–94).

**TABLE 6:** Estimated programmatic costs and projected long-term savings for high-impact human immunodeficiency virus (HIV) interventions such as dolutegravir (DTG)-based antiretroviral therapy (ART) rollout, voluntary medical male circumcision (VMMC) scale-up, and targeted pre-exposure prophylaxis (PrEP) distribution, based on real-world and modeling data from national programs in Africa and Southeast Asia.

Intervention	Country/region	Time frame	Total estimated cost	Infections/deaths averted	Net savings
DTG rollout (SSA)	22 countries	20 years	Baseline ART budget	131,000 deaths	$860M saved
VMMC scale-up	South Africa	15 years	$1.4 billion	>1 million	Cost effective
PrEP scale-up (AGYW)	Kenya	10 years	$50–100 million	20,000–30,000	Conditional
CAB-LA PrEP (injectable)	Uganda, Kenya	Pilot	TBD	High impact	Unclear affordability
NSP + OST	Belarus, Tajikistan	5–10 years	∼$2M–$5M	30%–50% transmission reduction	Cost effective
UTT + CHW linkage	Zambia, SA	5 years	$20–30/person/year	Large QALY gain	Economically justified
mHealth adherence	Global	3–5 years	$3–10/patient/year	Fewer treatment failures	High ROI
Pediatric ART access	SSA	Ongoing	$50–150 million	Lives saved + prevention	Long-term savings

## 7 Challenges and gaps in pharmacoeconomic research in HIV

Even with tremendous improvements in the prevention and treatment of HIV, important challenges and gaps in pharmacoeconomic study persist to restrict its real-world application to health decision-making worldwide. One of the main obstacles is dependence on mathematical modeling and controlled clinical trial data, which rarely reflect the realities of actual health systems, particularly in low- and middle-income settings where adherence variations, supply chain reliability, and local health infrastructure can have a profound impact on program performance. Evidence has increasingly shown that incorporating real-world implementation data into economic assessments can enhance their accuracy and relevance to policy (95). Moreover, large heterogeneity in treatment expenditure between settings, which is guided by delivery models and procurement systems, makes findings difficult to generalize. Most economic analyses still tend to concentrate primarily on direct medical expenses, without reference to indirect expenses like transport, lost wages, and wider household social implications, potentially resulting in underestimation of the real cost of HIV care. The other persistent gap is the underrepresentation of prevention measures such as PrEP, VMMC, and harm reduction interventions. These programs are plagued by their complex behavioral and structural dimensions, which are hard to model but are crucial for decreasing long-term incidence and linked treatment costs (96). In addition, conventional pharmacoeconomic strategies focus on aggregate cost-effectiveness without properly addressing equity. Interventions that are cost-effective at the population level can exclude important groups who are marginalized, such as transgender individuals, sex workers, and teenagers, further entrenching disparities (23). An increasing awareness of the importance of integrating distributional cost-effectiveness analyses that take into consideration social justice and equity perspectives in health economic assessments exists (97). Long-term sustainability is still not properly addressed. Whereas most studies concentrate on short- or medium-term expenditure, few fully take into account threats to donors’ dwindling support and shift to domestic funding. This poses issues of long-term sustainability of ART programs and prevention services in the future (98). Another key omission is the narrow inclusion of indirect benefits to society, such as enhanced workforce productivity and economic resilience, due to quantitative methodological difficulties in measuring these outcomes (99). In addition, new technologies such as long-acting injectable ART and broadly neutralizing antibodies raise new economic evaluation issues due to scarce real-world cost data and uncertain adherence patterns (100). Closing these research gaps will necessitate greater investment in implementation science, creation of standardized frameworks for incorporating social and equity effects, and more robust data infrastructure in order to support more sophisticated economic analyses. Sealing these gaps will ultimately increase the value and effectiveness of pharmacoeconomic analyses, so that HIV programs would prove to be cost-effective and socially equitable in the long run.

### 7.1 Equity, marginalized populations, and distributional cost-effectiveness in HIV pharmacoeconomics

Despite significant advances in economic evaluation, traditional pharmacoeconomic frameworks often inadequately capture the lived realities and structural inequities experienced by marginalized populations. Groups such as men who have sex with men (MSM), adolescent girls and young women (AGYW), female sex workers (FSW), transgender individuals, and people who inject drugs (PWID) frequently face disproportionate HIV risk but have lower access to prevention, testing, and sustained treatment. These disparities are shaped by criminalization, stigma, gender-based violence, socioeconomic marginalization, and health system discrimination factors that fall outside the narrow cost–effectiveness lens typically used in economic models.

Conventional cost-effectiveness analysis prioritizes maximizing aggregate health gains, which may obscure inequities if interventions benefiting low-risk or better-served populations appear more cost-saving at the population level. To address this gap, distributional cost-effectiveness analysis (DCEA) integrates equity weights and evaluates how health gains, financial risk protection, and opportunity costs are distributed across subgroups. Applying DCEA to HIV programs can reveal that interventions targeting marginalized communities may be less “efficient” in strict ICER terms yet deliver substantially higher social value when considering fairness, reduction of disparities, and ethical allocation principles.

For example, PrEP for AGYW or transgender women may have higher ICERs than PrEP for serodiscordant couples; however, DCEA demonstrates that prioritizing these groups prevents inequitable widening of HIV incidence gaps. Similarly, harm-reduction interventions such as NSPs and OST may appear modestly cost-effective from a health-system perspective but become dominant when incorporating avoided incarceration, reduced overdose deaths, and enhanced social inclusion. Equity-informed evaluations acknowledge the additional societal benefit of reaching populations historically excluded from HIV services and highlight the importance of resource allocation approaches that do not reinforce structural vulnerabilities. Integrating equity explicitly into pharmacoeconomic analyses strengthens policy relevance, particularly in settings where health systems must balance efficiency with rights-based commitments. Future evaluations should report distributional impacts, apply equity weighting, and include qualitative implementation barriers (e.g., stigma, criminalization, gender norms) that shape real-world cost and effectiveness profiles. By combining economic efficiency with ethical stewardship, equity-oriented pharmacoeconomics ensures that HIV investments contribute to both epidemic control and social justice (101).

## 8 Conclusion

Pharmacoeconomic evidence remains essential for optimizing HIV prevention and treatment strategies, particularly in settings where resources are constrained and programmatic decisions must balance effectiveness with affordability. This review demonstrates that while numerous interventions including ART, PrEP, VMMC, and harm-reduction programs show strong economic value, their cost-effectiveness is highly dependent on epidemiological context, adherence patterns, drug pricing, and time horizon selection. Considerable variability in modeling approaches, especially between Markov and dynamic transmission models, further underscores the need for methodological transparency and standardized analytical frameworks to ensure comparability across studies. A central finding of this review is that equity considerations are often insufficiently integrated into economic evaluations. Marginalized populations such as MSM, AGYW, transgender individuals, and PWID continue to face disproportionate burden yet remain underserved in many analyses. Incorporating distributional cost-effectiveness analysis (DCEA) and applying explicit equity weights can better capture the social value of interventions targeted to high-risk or structurally disadvantaged groups. Such approaches ensure that economic efficiency does not overshadow ethical imperatives or exacerbate existing disparities. Overall, the evidence affirms that strategic investment in HIV programs remains both cost-effective and socially beneficial. However, maximizing real-world impact requires economic evaluations that combine rigorous modeling, transparent reporting, and explicit attention to equity. Strengthening these dimensions will enable policymakers to design HIV responses that are not only economically sound but also aligned with principles of fairness, inclusion, and long-term epidemic control.
